# Training in obstetric and neonatal emergencies in Mexico: effect on knowledge and self-efficacy by gender, age, shift, and profession

**DOI:** 10.1186/s12909-020-02005-8

**Published:** 2020-03-31

**Authors:** Jimena Fritz, Alejandra Montoya, Héctor Lamadrid-Figueroa, Delia Flores-Pimentel, Dilys Walker, Sandra Treviño-Siller, Dolores González-Hernández, Laura Magaña-Valladares

**Affiliations:** 1 Dirección de Salud Reproductiva, Centro de Investigación en Salud Poblacional, Instituto Nacional de Salud Pública, Universidad No. 655, Santa María Ahuacatitlán, 62100 Cuernavaca, Morelos, CP Mexico; 2 Department of Obstetrics, Gynecology & Reproductive Sciences, Bixby Center for Global Reproductive Health, University of California San Francisco, 1001 Potrero Ave, San Francisco, CA 94110 USA; 3 Dirección de Retos y Determinantes del Sistema de Salud, Centro de Investigación en Sistemas de Salud, Instituto Nacional de Salud Pública, Universidad No. 655, Santa María Ahuacatitlán, 62100 Cuernavaca, Morelos, CP Mexico; 4 Association of Schools and Programs of Public Health (ASPPH), 1900 M St NW Suite 710, Washington, DC 20036 USA

**Keywords:** Emergencies, Obstetrics, Neonatal, Training, Health personnel, Simulation

## Abstract

**Background:**

Continuing education is essential for healthcare workers. Education interventions can help to maintain and improve competency and confidence in the technical skills necessary to address adverse events. However, characteristics of the health provider such as age (related to more critical and reflexive attitude); sex (relationship with gender socialization), profession and work conditions might have an influence on the effect of continuing education efforts.

**Methods:**

A training in the management of obstetric and neonatal emergencies (PRONTO, Spanish acronym for *Neonatal and Obstetric Rescue Program: Optimal and Timely treatment*) was implemented in 14 hospitals in six Mexican states between 2013 and 2014, with a before-after evaluation design. A total of 351 health providers including physicians, interns, nurses and midwives completed the training and were included in the analytic sample. Mixed-effects regression models were fitted to model changes in knowledge and self-efficacy scores after the training for each training topic. Interaction terms of training with age, gender, profession, and shift were included to evaluate possible heterogeneities of effect. All models considered the within-hospital clustering of participants.

**Results:**

After training, all participants showed a significant knowledge gain by an average of 19 percentage points for hemorrhage, 23 for neonatal resuscitation, 19 for shoulder dystocia, and 15 for preeclampsia/eclampsia (*p* < 0.001). Participants who worked night shifts showed lower scores for overall knowledge, compared with morning shift workers. Interns perceived the lowest self-efficacy while they scored very high in knowledge. Self-efficacy in managing obstetric and neonatal emergencies increased significantly by 16 percentage points in average.

**Conclusions:**

Our results show that PRONTO is generally successful in increasing knowledge and self-efficacy on all topics but knowledge and self efficacy levels vary greatly by factors such as work shift. Training should be particularly aimed at personnel working during weekends and night shifts, as well as interns and nurses.

## Background

Continuing education is essential for healthcare workers [1]. Education interventions can help to maintain and improve competency in the technical skills necessary to address adverse events [2]. However, knowledge and skills are not sufficient as it has been documented that up to 75% of medical mistakes occur because of the inability of professional teams to adequately respond to emergency situations, influenced by poor communication and a lack of planning [3].

Traditional teaching models in the medical environment emphasize hierarchies and the segmentation of education by profession or specialty, leading to a lack of effective communication and reduced patient safety [4–6]. In contrast, emergency care in real settings is provided by multidisciplinary teams that should be regularly trained together [7, 8].

Previous studies suggest that several interrelated factors influence learning and change of knowledge, such as: individual learning style; content of the educational intervention; management of the intervention; personal characteristics (both of participants and of the facilitators of the “new” knowledge); and the work and cultural environment in which the new knowledge must be implemented [9, 10].

However, not all individuals learn in the same way, and in addition to different types of learning (activist, reflexive, theoretical, and pragmatic) social determinants such as age (related to more critical and reflexive attitude) and sex (relationship with gender socialization) also have an influence [11]. Differences in attitudes to learning among men and women have been studied [12–14]. The socialization of gender means that women, who are often socialized from an early age to obey and accept new conditions, tend to internalize and anchor the importance of accomplishing and doing things right. Therefore, women tend to have better academic performance, and are more likely to integrate new knowledge and develop change quickly (not just in academic matters) [14].

Age-based differences in learning have also been studied [15]. At an older age, it is harder to learn and difficult to accept and implement new things. This is because individuals have developed skills over years; unlearning what has previously been learned is challenging. Older individuals also tend to be more critical and reluctant to integrate new knowledge. However, as the type of learning at this stage is more critical and rational, providing opportunity to discuss and put new knowledge into practice makes it feasible to achieve change in practice [15–17].

Stress and motivation are thought to impact learning. Simulation-based training in emergencies produces stress, but helps learning; however, the degree of intrinsic motivation of healthcare providers is fundamental to such learning [18].

In 2009, the National Institute of Public Health of Mexico (INSP, Spanish acronym for *Instituto Nacional de Salud Pública*), in collaboration with researchers from the University of Washington, the University of Utah, and the University of Maryland, developed PRONTO (Spanish acronym for *Neonatal and Obstetric Rescue Program: Optimal and Timely treatment*), an obstetric and neonatal emergency training program based on simulations and team work [19]. PRONTO was piloted in five hospitals in Mexico during 2009–2010. It was found that the training learning modalities were accepted and participants’ knowledge, self-efficacy, and teamwork were improved [19]. From 2010 to 2015, the impact of the program was evaluated in a pair-matched, hospital-based controlled trial involving 12 hospitals in three Mexican states. Both process and impact indicators showed positive results in terms of increased knowledge and self-efficacy and decreased rates of cesarean sections and neonatal mortality [20, 21]. In light of the favorable results, the Ministries of Health of a number of Mexican states asked for more PRONTO trainings to be performed, of which data were recorded and analyzed in the present study.

This study aimed to evaluate the effect of PRONTO on knowledge and self-efficacy on obstetric and neonatal emergency care among health providers, using a before-after study design. Pre-existing differences in knowledge and self-efficacy, as well as heterogeneous effects of the training by gender, age, shift, and profession were also evaluated.

## Methods

### Intervention

The PRONTO intervention has been described in detail elsewhere [19–22]. In brief, the PRONTO training is based on clinical cases, interactive exercises, and communication practices, using an interprofessional approach. The training has minimal didactic content; most teaching occurs through interactive team-building exercises, targeted skills sessions, highly realistic simulations of obstetric and neonatal emergencies, and video-guided debriefings immediately following each scenario. Childbirth and postpartum care simulations, both with and without complications, are conducted. Training sessions are led by a team of nurse midwives, nurses and physicians including at least one PRONTO master trainer and 3–4 local team members that have completed a PRONTO train-the-trainer course. Training occurs in a real work environment (trainees are mixed groups comprised of physicians, nurses and midwives), using resources that are usually available at medical units, providing a highly realistic environment. Simulations are conducted in the emergency area and in labor, delivery, operation, and recovery rooms. PartoPants™, which are hybrid birth simulators made by modifying recycled surgical scrubs, are worn by a participant who plays the role of the patient during an obstetric emergency. A Laerdal NeoNatalie© simulator is used to practice neonatal resuscitation. Participants assume roles according to their profession and work area, therefore rendering a more realistic training experience. The simulations are video recorded for subsequent feedback involving the whole group, which favors constructive learning and the detection and avoidance of potential mistakes [20].

The intervention comprises two modules: Module I (MI: 16 h, conducted over two consecutive days – 8 h each day) is focused on obstetric hemorrhage (OH), neonatal resuscitation (NR), team work, and communication skills. Module II (MII, 8 h, conducted over 1 day) is scheduled 3 months after MI and reinforces the topics in MI, as well as addressing preeclampsia/eclampsia (PE) and shoulder dystocia (ShD) [20]. All training sessions were implemented from 9 AM to 5 PM during weekdays, even for afternoon, night and weekend shift workers.

### Participants

The State Ministries of Health and Institutes for Women requested the training to be implemented in 14 Ministry of Health-run hospitals in the Mexican states of Guerrero, Morelos, Oaxaca, Puebla, Quintana Roo, and Veracruz, which provide care for mostly low socioeconomic level population without access to social security. Teaching coordinators or hospital directors were advised by the trainers to invite personnel from different disciplines who worked in childbirth and neonatal care or who were otherwise involved in obstetric emergency care, although each hospital had the final call on who was to attend. Most participants were obstetrician-gynecologists (OBGYN), pediatricians, general practitioners, interns, nurses, or professional midwives. An oral consent letter was read to the potential participants at the beginning of the training. All providers in attendance were offered the option to receive the training regardless of their willingness to participate in the before-after evaluation (and hence the study).

Sample size. A total of 351 participants gave consent to participating in the study and completed pre and post questionnaires, while 55 persons only completed pre-questionnaires and were excluded. According to data from our study, assuming a before-after correlation of 0.56 a pre-treatment mean of 50 and a common standard deviation of 15 points, the minimum detectable effect size with the *n* = 351 sample size and a power of 80% was 2.1 percentage points in the case of knowledge. In the case of self-efficacy, assuming a before-after correlation 0.5, a pre-treatment mean of 80 and a standard deviation of 11 pre-test and 17 post-test, the minimum detectable effect size with a power of 80% was 2.24 percentage points.

### Study variables

Outcomes: Knowledge & Self-efficacy.

Participants completed pre- and post-training questionnaires immediately before and after each module, evaluating knowledge of evidence-based practices in identifying, preventing, and managing obstetric and neonatal emergencies as well as participant confidence in his/her own ability to perform key skills (self-efficacy). The questionnaires were a revised version of those used in the 2010 PRONTO pilot [19]. The self-efficacy scales were based on the model developed by Bandura [23]. Self-efficacy is defined as the sense of security each person experiences in relation to his/her ability to perform the necessary actions during emergencies. Following the standard methodology for measuring self-efficacy, individuals were presented with items portraying different levels of task demands, and they rated the strength of their belief in their ability to execute the needed procedures. In total, the evaluation instrument contained 26 knowledge questions and 27 questions on self-efficacy in five categories: neonatal resuscitation, obstetric hemorrhage, general obstetric emergency, shoulder dystocia, and preeclampsia/eclampsia.

In the case of the knowledge questions, participants’ responses were coded as correct or incorrect, then we obtained a knowledge score (both total and for each category) consisting on the percentage of correct answers by each particular individual in the sample. In the case of self-efficacy items, the participants rated themselves on a scale of 0–100 in which 0 means complete lack of confidence and 100 means total confidence; in this case we defined the self-efficacy score as the arithmetic mean of the participant’s answers, both total and by category.

### Covariates

Shift. This variable included information on the work shift of each particular participant, classified as morning, afternoon, night or weekend/holidays. In Mexico, health workers are typically assigned to only one shift, and, although it is theoretically possible to change to a different one, it is difficult to do so and workers tend to remain working in that particular shift indefinitely. Of course, this does not apply to interns, who are at the hospital at all times; in this case we arbitrarily defined them as belonging to the morning shift as in Mexico each intern spends all morning shifts in the hospital, and only covers afternoon, night and holiday/weekend shifts every three to four days (we could say interns work predominantly in the morning). Profession. Participants in the training were classified according to their profession as general practitioners, obstetricians, pediatricians, medical interns, other medical specialists, nurses, obstetric nurses and midwives.

We use the term “nurses” to refer to general nurses; nurse students were included in this category; the term “obstetric nurses” is used to refer to nurses who are certified in obstetric care. We use the term “midwives” to refer to staff that have completed either technical training or a bachelor’s degree in midwifery. As only one obstetric nurse was trained, we decided to reclassify her in the same category as midwives.

Other covariates. Additional covariates included in the model were self-reported gender and age of the participant.

### Statistical analyses

Analyses were conducted in three phases. First, baseline participants’ characteristics were described in terms of means and proportions. Secondly, we compared the participants’ characteristics to those who only completed baseline questionnaires but eventually dropped out of the training using a logistic regression model with exclusion of the final sample as the outcome and gender, age, work shift and profession as covariates. Lastly, we modeled knowledge and self-efficacy as a function of participant characteristics and the PRONTO training, both overall and by training topic.

To identify factors related to knowledge and self-efficacy and estimate the before-after changes in outcomes, a set of longitudinal linear regression models with mixed effects (random effect at the individual level) was fitted to the data; the model was used both to estimate the influence of covariates on baseline knowledge and self-efficacy and to model changes in the outcomes after the training using a complete-case approach. All models considered the clustered structure of participants by hospital by including a fixed effects term of the hospital [24]. Models of knowledge and self-efficacy were fitted for each training topic, as well as the whole course average. In all cases, the outcome variable was the knowledge or self-efficacy score obtained (in percentage points), and the main independent variable was a dummy indicating time (i.e., before or after the intervention). We evaluated the effect of gender, age (categorized as < 30, 30–49, and > 50 years), shift during which the participants worked (morning, afternoon, evening, and weekend/holidays), and profession (nurses, midwives, interns, general practitioners, pediatricians, OBGYN, and other medical specialties). The self-efficacy models included the knowledge score in the same topic as a covariate. Interaction terms for time (dummy variable) with gender, age, shift, and profession were included to evaluate possible heterogeneities of effect. The analyses were performed using Stata version 13.0 (StataCorp LP, College Station, TX, USA).

## Results

From June 2013 to January 2014, 351 healthcare providers from 14 hospitals in six Mexican states received at least one of the two PRONTO training modules and completed pre and post knowledge and self-efficacy evaluations; of these 205 participants attended both modules, 92 attended only module I and 54 attended only Module II.. The majority of participants were women (66.8%), aged 16–69 years (average age 39 years). The largest professional group was nurses (45.9%), followed by general practitioners (24.8%), and OBGYN (12.5%); the remaining 19.4% included obstetric nurses and midwives, interns, pediatricians, and other medical specialists. Overall, 48.6% of participants worked morning, 17.3% afternoon, 18.4% night shifts, and 15.2% on weekends and holidays (Table 1). The 55 health providers that dropped out of the training did not show any significant differences in terms of neither baseline knowledge nor self-efficacy relative to the participants, however they were significantly more likely to be male (adjusted OR for drop-out 2.77, *p* = 0.02) and to be doctors rather than nurses or midwives (OR = 5.44, *p* < 0.01) (Additional Table 1).

**Table 1:** Distribution of age, gender and working shift of PRONTO training participants, by profession. 2013–2014

	Age		Gender			Shift				
			All	Male	Female	All	Morning	Afternoon	Night	Weekend/Holiday
	***n***	Mean	***n***	***n***	***n***	***n***	***n***	***n***	***n***	***n***
		SD		%	%		%	%	%	%
**Profession Obstetrician-gynecologists**	44	43.7	44	26	18	40	26	4	5	5
		10.6		59.1	40.9		65	10	12.5	12.5
**Nurses**	159	38.1	157	14	145	155	79	30	34	12
		9.4		8.8	91.2		51.0	19.4	21.9	7.7
**Midwives / obstetric nurses**	7	32.4	7	0	7	7	5	1	1	0
		12.5		0	100		71.4	14.3	14.3	0.0
**Interns**	7	24.3	7	1	6	7	7	0	0	0
		1.4		14.3	85.7		100	0.0	0.0	0.0
**General practitioners**	87	38.0	86	49	38	77	31	15	17	14
		10.0		56.3	43.7		40.3	19.5	22.1	18.2
**Pediatricians**	14	39.6	14	8	6	13	3	4	1	5
		10.6		57.1	42.9		23.1	30.8	7.7	38.5
**Other medical specialists**	31	36.8	31	18	13	30	14	3	4	9
		9.8		58.1	41.9		46.7	10.0	13.3	30.0
**Missing data**	2	–	5	–	–	32	–	–	–	–
		–					–	–	–	–
**Total**	351	38.3	351	116	233	351	160	57	62	50
		10.1		33.2	66.8		48.6	17.3	18.8	15.2

Baseline (pre training) knowledge and self-efficacy assessment scores according to descriptive characteristics are shown in a supplementary file (Additional Table 2). After adjustment for covariates no significant differences were observed between men and women. However, women perceived less self-efficacy in obstetric emergencies than men. A greater age was associated with lower average knowledge scores for OH and NR, but it was not significantly related to knowledge of PE and ShD. Participants aged 30–49 years perceived less self-efficacy than those younger than 30 years (Table 2 and Additional Table 3).

**Table 2:** Adjusted knowledge and self-efficacy average score differences at baseline (pre-training), by participants’ characteristics

		Knowledge		Self-efficacy	
		***n*** = 347		***n*** = 347	
		Coef	95% CI	Coef	95% CI
**Sex**	Male (ref)	–		–	
	Female	0.09	(−3.12,3.29)	−4.02**	(−7.80,-0.23)
**Age (years)**	< 30 (ref)	–		–	
	30,49	−2.02	(−5.34,1.31)	4.24**	(0.24,8.24)
	50+	−5.65**	(−10.03,-1.27)	1.84	(−3.48,7.163)
**Profession**	OBGYN (ref)	–		–	
	Nurses	−18.84***	(−23.27,-14.41)	−3.58	(−9.24,2.09)
	Midwives / obstetric nurses	−12.25**	(−22.14,-2.35)	2.78	(−9.54,15.10)
	Interns	−8.62	(−19.04,1.81)	−17.81***	(−29.91,-5.71)
	General practitioners	−7.20***	(−11.74,-2.67)	0.62	(−4.79,6.02)
	Pediatricians	−1.5	(−8.89,5.89)	−5.5	(−14.22,3.22)
	Other medical specialists	−9.25***	(−14.52,-3.98)	1.83	(−4.46,8.12)
**Working shift**	Day (ref)	–		–	
	Afternoon	−2.02	(−5.34,1.31)	−0.63	(−5.18,3.92)
	Night	−5.65**	(−10.03,-1.27)	−2.34	(−6.76,2.08)
	Extended (weekends and holidays)	0.09	(−3.12,3.29)	−2.17	(−6.77,2.43)

*Coef* Coefficient from mixed-effects regression models, *CI* confidence interval. *** *p* < 0.01, ** *p* < 0.05

Profession was strongly associated with knowledge scores. Compared with OBGYN, all participants had significantly less knowledge about OH and ShD. Similar findings were observed for PE, except for interns and other medical specialists. Nurses had significantly less knowledge of NR than OBGYN, and pediatricians showed the highest scores (Additional Table 3). Pediatricians and general practitioners were the only cadres who had average scores for obstetric emergencies that did not significantly differ from those of OBGYN. Participants who worked night shifts showed lower scores for overall average knowledge (including OH and NR) than those who worked morning shifts. There were no significant differences among participants who worked weekends and holidays or afternoon shifts relative to morning shift (Table 2) Interns were the only cadre that perceived a significantly lower self-efficacy compared with OBGYN. In general, OBGYN had the highest perception of self-efficacy for emergency care except for NR, where all other cadres showed higher perceived self-efficacy than OBGYN (Table 2 and Additional Table 3).

**Fig. 1:**
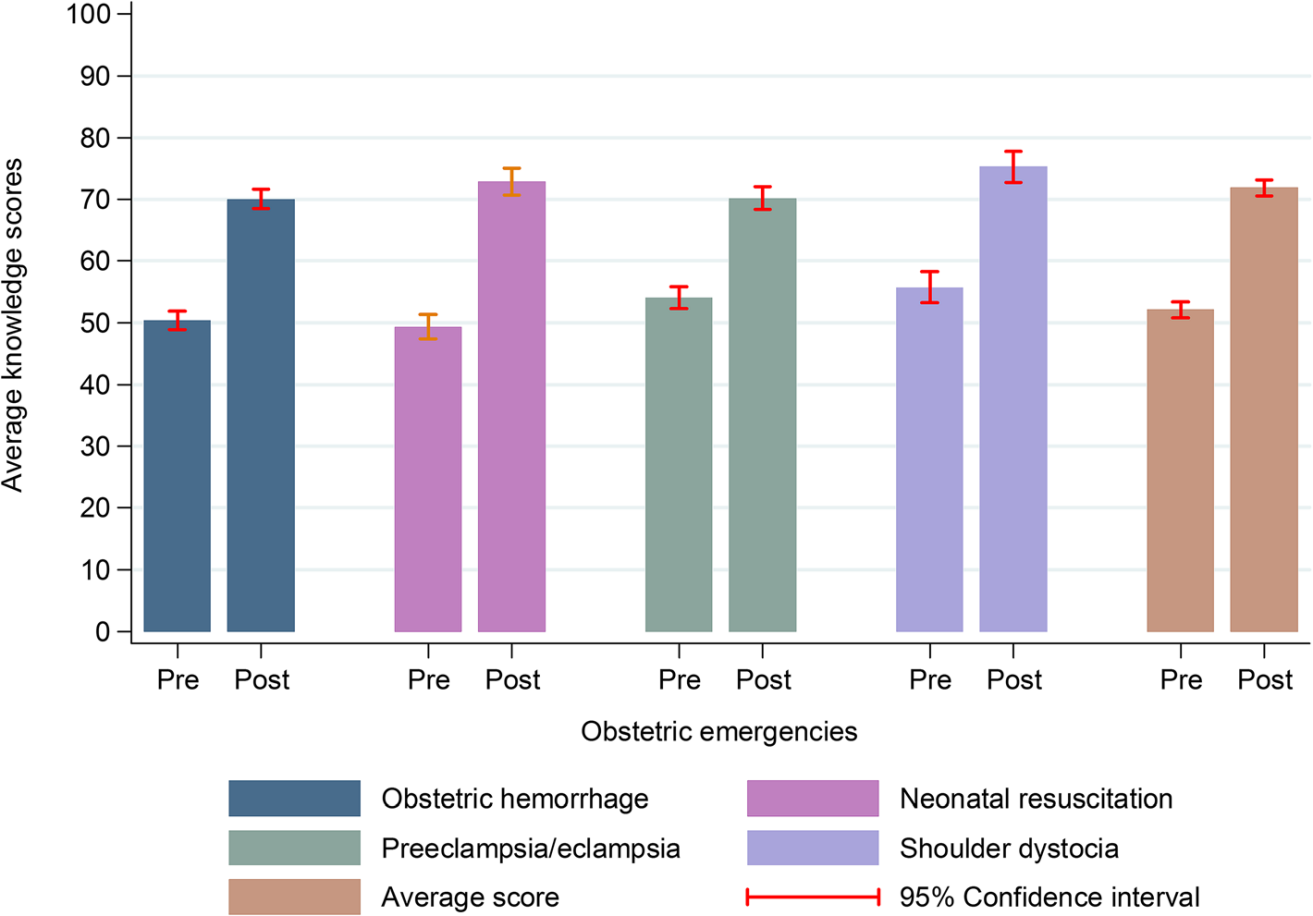
Before-after training change in average knowledge scores, by course subject

After training, all participants showed a significant knowledge gain, independent of personal characteristics and topic (Table 3). Nurses, who started the training program with the lowest scores, obtained the greatest benefit (Table 3). The highest knowledge gain was observed on NR with 23 percentage points (*p* < 0.001), followed by OH and ShD with 19 percentage points and PE with 15 percentage points (*p* < 0.001) (Fig. 1).

**Table 3:** Change in average knowledge and self-efficacy scores after PRONTO training, by participants’ characteristics

		Knowledge		Self-efficacy	
		***n*** = 347		***n*** = 347	
		Change	95% CI	Change	95% CI
**Gender**	Male	20.1	(17.4,22.8)	3.9	(0,7.8)
	Female	19.8	(18,21.6)	6.1	(3,9.3)
**Age (years)**	< 30	19.8	(16.8,22.8)	8.7	(4.5,12.8)
	30,49	20.8	(19,22.6)	4.2	(1,7.4)
	50+	16.4	(12.8,20.1)	4.3	(−0.7,9.4)
**Profession**	OBGYN	16.6	(12.5,20.6)	6.6	(1.3,11.8)
	Nurses	22.0	(19.8,24.3)	5.6	(1.8,9.3)
	Midwives / obstetric nurses	15.9	(6.1,25.7)	0.0	(−13.7,13.7)
	Interns	16.9	(6.1,27.7)	16.9	(4.1,29.7)
	General practitioners	18.7	(15.7,21.6)	3.6	(−0.5,7.7)
	Pediatricians	16.0	(9, 23)	8.00	(− 0.9,16.9)
	Other medical specialists	20.6	(16,25.1)	4.1	(−1.8,10.1)
**Working shift**	Day	19.7	(17.7,21.7)	4.8	(1.6,8)
	Afternoon	19.0	(15.6,22.3)	5.3	(0.5,10.2)
	Night	22.3	(19,25.5)	6.1	(1.4,10.8)
	Extended (weekends and holidays)	18.7	(14.9,22.4)	6.0	(1,10.9)

Mean changes estimated by mixed-effects linear regression models, including interaction terms for each participant’s covariates, *CI* confidence interval

**Fig. 2:**
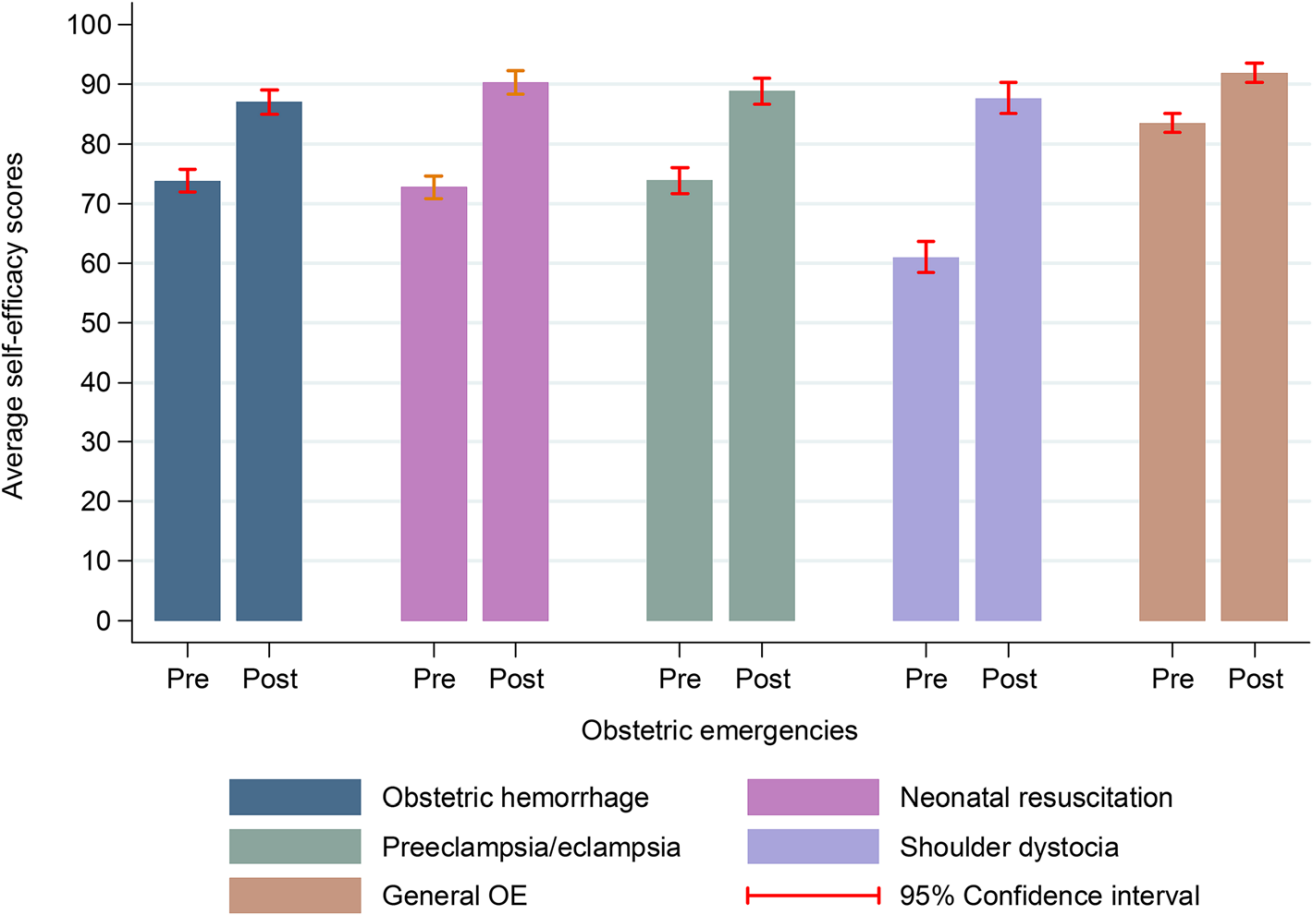
Before-after training change in average self-efficacy scores, by course subject

For self-efficacy, we also found a gain in every topic, regardless of participants’ characteristics (Fig. 2 and Table 3); the average increase was 16.1 percentage points. The largest increase in self-efficacy for performing clinical procedures was 26.6 percentage points in the case of ShD (Additional Table 4). Obstetric emergencies was the topic that showed the smallest increase, although the difference was statistically significant (8.4 percentage points; *p* < 0.001) (Fig. 2).

## Discussion

The PRONTO program increased knowledge and self-efficacy in obstetric and neonatal emergencies among healthcare providers. This is consistent with results obtained in previous studies [20, 21, 25]. Our analysis showed a significant increase in knowledge and self-efficacy in every course topic; this is a relevant result given that these complications are among the main causes of maternal and neonatal mortality in Mexico [26].

One of the most important findings of our study is the differences in knowledge and self-efficacy by gender, age, shift, and profession. Compared with OBGYN, all health professionals had less knowledge of OH, PE, and ShD. Although this was expected, the results were heterogeneous according to the outcome: interns had the lowest average self-efficacy of all cadres, while paradoxically they scored high in knowledge, only behind pediatricians and OBGYN and above general practitioners and other specialists (Table 1). Paradoxically, medical interns where the only cadre that scored a significantly lower baseline self-efficacy compared to OBGYN (Table 2). This is interesting because as students, they are generally reluctant or lack the confidence to defy their superiors in a hierarchical medical setting even though they might actually be more knowledgeable [6]. Although self-efficacy in performing clinical procedures increased by 8.6–26.6 percentage points in general, women and those aged 30–49 years perceived less self-efficacy on average.

Another interesting result was that professionals who worked evening shifts had less knowledge in NR than those who worked morning shifts. Night shift workers showed lower knowledge overall and in both OH and NR. Previous studies have shown that night shifts pose more risk to patients [27], and that quality of healthcare varies between shifts, with night shifts and weekends being the most unprotected [28]. We found that the number of participants who worked the night shift (18.4%) was disproportionately small compared to morning shift workers (48.6). This trend appears to repeat itself in other trainings by our research team and may signal a systematic lack of training/continuous education aimed at this group, although detailed studies on this particular matter are merited.

Analysis of variables for potential heterogeneous effects (age, shift, gender, and profession) showed that nursing staff gained an average of four more points in knowledge than medical staff (including general practitioners and specialists). However, at the beginning of the intervention nurses had the lowest score and therefore a larger scope for improvement. Of note was the performance of professional midwives in terms of knowledge of the management of obstetric emergencies. Midwives’ average score was much higher than that of nurses, and was close to that of general practitioners at baseline (52% and 56%, respectively, compared with 44% for nurses). However, midwives’ knowledge gains during training were smaller than those of general practitioners (16 vs. 19 percentage points, respectively). Although these results are interesting, the reduced sample size (*n* = 7 midwives) prevented statistical significance and should be interpreted with caution.

The simulations were conducted in situ (i.e., in the same place where staff provides healthcare), which gave participants a clear view of the organizational barriers that are usually encountered when responding to an emergency, and added realism to the simulation because the scenario occurred where childbirth and obstetric/neonatal emergency care is provided [29–31]. During the simulations, favorable actions were taken to preserve patient safety; for example, during a convulsive crisis, magnesium sulphate was administered quickly. This is consistent with other studies using simulations that found favorable actions were taken not only after transmitting knowledge, but also after its implementation through simulation [29]. These results are consistent with other approaches that combined interactive activities, review of case reports, simulations, and feedback [32–35].

Previous authors [29] noted that the most necessary elements for successful medical trainings are simulations and repeated training periods. We recommend ongoing interprofessional training in all medical units to maintain the required knowledge and skills as the literature suggests that knowledge in adults might be diluted 3–6 months after training [36] although recent research on obstetric care puts this figure up to 12 months [25], suggesting repeated drills might be beneficial to ensure retention.

The PRONTO program contains elements that enhance interprofessional team work by introducing communication concepts based on the TeamSTEPPS program [30], which is designed to preserve patient safety. Better integration of obstetric care staff is important, because this changes the paradigm of medical teaching and focuses on training teams [37]. However, barriers preventing the provision of resources, equipment, effective communication, team work, and leadership may be deciding factors for the medical personnel to address emergency situations in a timely manner for both the mother and newborn.

### Limitations

This study has several limitations worth considering. Firstly, the lack of a comparison or control group means that we cannot be certain the observed changes are fully attributable to the intervention, or rather to clinical experience/knowledge gained by other means in the time between evaluations, although this is unlikely as only 1 day passed between the pre and post tests. Several barriers interfered with the implementation of the program, including location changes for training (due to the remodeling of certain hospital centers). Invitations to the training were not in charge of the researchers but rather occurred via INSP–Ministry of Health–hospital directors-participants. Unfortunately we did not gather information that would allow us to ascertain whether participants were truly involved in obstetric and neonatal care and to what extent. The limitations on the external validity of the present findings are also worth mentioning, as neither hospitals nor participants were randomly selected. Another limitation is our lack of information on whether participants who worked the night shift were sufficiently rested the night before the evaluation, as this might have hindered their ability to grasp the course contents; as such our finding regarding lower knowledge for participants during the night shift might be biased.

Regarding the arbitrary allocation of interns to the morning shift, the number of interns that participated in the study was quite small (*n* = 7), comprising only 2% of the sample at baseline, so it is unlikely that any induced bias is large. However, in order to have a more objective appraisal of any potential bias due to the assignment of interns to the morning shift, we re-estimated knowledge score averages excluding them and the resulting change in the estimates was negligible (coefficients vector Pearson’s *r* = 0.992).

A considerable number of health providers that completed the pre-questionnaire were excluded as they did not complete the post questionnaire. As the drop-out seems to be unrelated to the study outcomes but rather dependent on observed covariates, the potential for bias is small [38]. Regardless, the finding that male doctors were generally more reluctant to participate in the course calls for in-depth qualitative studies on the role of gender on obstetric healthcare and on the willingness to engage in interprofessional trainings in general. Indeed, at least one study has identified power relationships as a barrier for interprofessional collaboration in healthcare [39].

Finally, we were not able to evaluate neither health outcomes, skills, nor the long-term retention of knowledge and self-efficacy. It is important to recognize self-efficacy was measured as a very short-term perception right after the trainings had finished and we were unable to observe how it related to actual performance in obstetric practice. Moreover, we are not aware of studies relating self-efficacy to actual skills. In spite of these shortcomings, PRONTO has previously showed impact on some health outcomes [21]. Similarly, a separate study on the effect of PRONTO training on good practices during delivery in Mexico showed the effect on participants is mostly retained at least after 12 months [40].

## Conclusions

The PRONTO training produced positive and significant results in terms of increased knowledge and self-efficacy in all topics. Low-tech, high-fidelity medical simulations and the interprofessional approach used in PRONTO training have repeatedly proven to be an effective teaching method in a hospital environment.

Changes in the study outcomes suggest that interns have less self-efficacy relative to their knowledge level. Nurses, midwives, interns and general practitioners benefitted the most from the program. It is important to promote self-efficacy among women, students, and young professionals to improve health outcomes for patients. Simulation-based education should complement continuous medical education for health professionals. Results from the present study highlight that continuing education and resources are indispensable for every health professional to improve health outcomes. Furthermore, interprofessional education should be favored to enhance communication and team work among healthcare professionals, particularly among those in charge of emergency management, avoiding neglecting training for night shift workers, nurses and interns.

## Supplementary information

**Additional file 1:Table 1.** Multiple logistic model of the determinants of study drop-out (0 = remained in the study; 1 = dropped out).

**Additional file 2:Table 2.** Baseline knowledge and self-efficacy of PRONTO training participants, 2013–2014.

**Additional file 3:Table 3.** Adjusted knowledge and self-efficacy score differences at baseline (pre-training), by topic and participants’ characteristics.

**Additional file 4:Table 4.** Change in average knowledge and self-efficacy scores after PRONTO training, by topic and participants’ characteristics.

## Data Availability

The datasets used and/or analyzed during the current study are available from the corresponding author on reasonable request.

## Abbreviations

PRONTO: Programa de Rescate Obstétrico y Neonatal Tratamiento Óptimo y Oportuno
OBGYN: Obstetrician-gynecologists
INSP: Instituto Nacional de Salud Pública
MI: Module I
OH: Obstetric hemorrhage
NR: Neonatal resuscitation
MII: Module II
PE: Preeclampsia/eclampsia
ShD: Shoulder dystocia
INMUJERES: Instituto Nacional de las Mujeres
